# Novel pharmacologic inhibition of lysine-specific demethylase 1 as a potential therapeutic for glioblastoma

**DOI:** 10.1038/s41417-024-00847-8

**Published:** 2024-11-05

**Authors:** Keiko Shinjo, Takashi Umehara, Hideaki Niwa, Shin Sato, Keisuke Katsushima, Shinya Sato, Xingxing Wang, Yoshiteru Murofushi, Miho M. Suzuki, Hiroo Koyama, Yutaka Kondo

**Affiliations:** 1 Division of Cancer Biology, Center for Neurological Diseases and Cancer, Nagoya University Graduate School of Medicine, Nagoya, Japan; 2 Laboratory for Epigenetics Drug Discovery, RIKEN Center for Biosystems Dynamics Research, Yokohama, Japan; 3 Morphological Information Analysis Laboratory, Kanagawa Cancer Center Research Institute, Yokohama, Japan; 4 Drug Discovery Chemistry Platform Unit, RIKEN Center for Sustainable Resource Science, Wako, Japan; 5 Center for One Medicine Innovative Translational Research (COMIT), Nagoya, Japan; 6 Institute for Glyco-core Research (iGCORE), Nagoya University, Nagoya, Japan

**Keywords:** Drug development, CNS cancer

## Abstract

Lysine-specific demethylase 1 (LSD1/KDM1A) is a pivotal epigenetic enzyme that contributes to several malignancies including malignant glioma. LSD1 is a flavin adenine dinucleotide dependent histone demethylase that specifically targets histone H3 lysine (K) 4 mono- (me1) and di-methylation (me2) and H3K9me1/2 for demethylation. Herein we report the development of an LSD inhibitor, S2172, which efficiently penetrates the blood-brain barrier. S2172 effectively suppresses LSD1 enzymatic activity, resulting in the depletion of cell growth both in vitro in glioma stem cells (GSCs) (mean half-maximal inhibitory concentration (IC_50_) of 13.8 μM) and in vivo in a GSC orthotopic xenograft mouse model. Treatment with S2172 robustly reduced the expression of the stemness-related genes *MYC* and *Nestin* in GSC cells. Consistent with this, chromatin immunoprecipitation-sequencing revealed a significant S2172-dependent alteration in H3K4me2/H3K4me3 status. Furthermore, we identified 284 newly acquired H3K4me2 peak regions after S2172 treatment, which were encompassed within super-enhancer regions. The altered H3K4me2/H3K4me3 status induced by S2172 treatment affected the expression of genes related to tumorigenesis. Our data suggest that targeting LSD1 with S2172 could provide a promising treatment option for glioblastomas, particularly due to targeting of GSC populations.

## Introduction

Malignant glioma is a highly aggressive brain tumor with a median overall survival of 14–17 months [1]. Recent advancements in genome sequencing studies have led to the classification of glioma into several subtypes based on the mutation status of *isocitrate dehydrogenase* (IDH) and *histone H3* genes, *telomerase reverse transcriptase* (*TERT*) promoter mutation, *epidermal growth factor receptor* (*EGFR*) amplification and 1p/19q deletion [2–4]. Despite significant progress in understanding glioma pathophysiology, alkylating agents remain the standard chemotherapy, and effective targeted therapies have yet to be identified for this disease [5]. Challenges associated with developing effective glioma treatments include tumor cell plasticity (which is epigenetically regulated) and phenotypic changes, such as the reversible transition between glioma stem cells (GSCs) and differentiated glioma cells [6, 7]. For successful elimination of malignant glioma cells (including GSCs), therefore, attention has been focused on therapeutic targeting of epigenetic regulators.

Lysine-specific demethylase 1 (LSD1), the first identified histone demethylase [8] is also known as KDM1A, AOF2, or BHC110. It is a flavin adenine dinucleotide (FAD)-dependent histone demethylase responsible for removing mono (me1)- and dimethyl (me2) groups from histone H3 lysine 4 (H3K4) and lysine 9 (H3K9) residues [9]. LSD1 functions in transcriptional repression as a component of the CoREST (co-repressor for element-1-silencing transcription factor) complex and the NuRD (nucleosome remodeling and histone deacetylation) complex by demethylating H3K4me1/me2 [10, 11]. LSD1 also demethylates H3K9me2 and H3K9me1 via interaction with nuclear receptors such as the androgen receptor and the estrogen receptor to promote gene activation [12]. Given its involvement in modifying two distinct histone marks— H3K4, an activating mark, and H3K9, a repressive mark—it is suggested that LSD1 finely tunes both the activation or repression of transcription [9].

Via its modulation of gene expression programs, LSD1 is an essential regulator of stem cell differentiation in both embryonic and adult cells. During development, perturbation of LSD1 function precipitates a cascade of differentiation deficits [13, 14]. Pertaining to malignancies, dysregulated expression of histone demethylases, including LSD1, is a common phenomenon across numerous cancer types [15]. Increased levels of LSD1 have been documented in diverse human tumors including prostate cancer [12], small cell lung cancer [16, 17], breast cancer [18], and hematopoietic malignancies [19]. In glioma cells, inhibition of LSD1 reduces signaling controlled by the stemness-associated gene *MYC*, which is regulated by super-enhancers [20]; indeed, expression of *MYC* itself is downregulated following LSD1 inactivation [21]. Currently, several LSD1 inhibitors are undergoing oncology clinical trials, which are primarily focused on patients with hematopoietic malignancies [22].

LSD1 expression is particularly upregulated in GSCs compared to differentiated glioma cells. Targeting LSD1 with our earlier LSD1 inhibitor, S2101 [23] reduces the viability of GSCs without affecting normal astrocyte viability [24]. Subsequently, we developed S2157, an LSD1 inhibitor capable of traversing the blood-brain barrier [25]. Indeed, S2157 induced apoptosis in T-cell acute lymphoblastic leukemia cells and efficiently eliminated central nervous system leukemia in a murine model [26]. In our current report, we describe the development of S2172, which is another LSD1 inhibitor derived from S2101. S2172 exhibits efficient brain penetration and a reduced inhibitory constant (*Ki*) compared to both S2101 and S2157. Comprehensive studies also show that S2172 exhibits robust antitumor efficacy against brain tumor cell lines both in vitro and in vivo.

## Materials and methods

### Chemicals

S2101 and S2157 were synthesized as previously described [23, 25]. S2172 was synthesized from S2101 [23] as a 1:1 mixture of *cis* and *trans* isomers with regard to the 1,4-diaminocyclohexyl moiety. The scheme of the synthesis of S2172 is shown in Supplementary Fig. S1A. Sodium triacetoxyborohydride (536 mg, 2.53 mmol) was added to a solution of S2101 (430 mg, 1.33 mmol), NaOAc (218 mg, 2.66 mmol), and 4-(tert-butoxycarbonylamino) cyclohexanone (283 mg, 1.33 mmol) in CH_2_Cl_2_ (7 mL) at room temperature. After the reaction mixture was stirred for 1 h, it was neutralized with 2 mol/L NaOH*aq* (3 mL). After dilution with CH_2_Cl_2_ (30 mL), it was washed with H_2_O (20 mL). The aqueous phase was extracted with CH_2_Cl_2_ (20 mL), and the combined organic phase was dried over anhydrous Na_2_SO_4_, filtered, and concentrated in vacuo. The crude mixture was dissolved in CH_2_Cl_2_ (13 mL). Triethylamine (0.37 mL, 2.7 mmol) and Boc_2_O (580 mg, 2.7 mmol) were added to the CH_2_Cl_2_ solution. The reaction mixture was stirred for 2 days, and then concentrated in vacuo. The crude product was purified using silica gel column chromatography (SiO_2_ 30 g) with Hexane-AcOEt as eluant to afford intermediate A (180 mg, 0.31 mmol, 23% from S2101). 4 N HCl/1,4-dioxane (3 mL) was added to the solution of intermediate A (180 mg, 0.31 mmol) in 1,4-dioxane (3 mL) and stirred for 1 day. The mixture was concentrated and dried in vacuo to yield the diHCl salt of S2172 (135 mg, 0.30 mmol, 23% from S2101) as a colorless powder.

^1^H NMR (DMSO-d6, 270 MHz) δ: 1.22–1.58 (6H, m), 2.01–2.02 (4H, m), 2.87–3.15 (4H, m), 4.99 (1H, A of ABq, J = 13.5 Hz), 5.10 (1H, B of ABq, J = 13.5 Hz), 6.72 (1H, d, J = 8.1 Hz), 7.22 (1H, m), 7.37 (3H, m), 7.54 (2H, m), 8.10 (3H, br m), 9.67 (2H, br m).

### Crystallographic analysis

Human LSD1 protein (residues 172–833) was prepared as described previously [23]. For co-crystallization, 54 µM (4 mg/mL) LSD1 solution was mixed with S2172 solution (in DMSO solvent) at a protein–inhibitor molar ratio of 1:5 and left overnight. LSD1–S2172 co-crystals were grown using the hanging drop method at 20°C, where the protein solution was mixed with an equal volume of reservoir solution containing 100 mM MES (pH 6.5), 200 mM diammonium tartrate, 500 μM TCEP, and 14% PEG3350. Small rod-like crystals appeared overnight and were harvested after a week. Crystals were flash-cooled with liquid nitrogen before data collection, using 20% glycerol as a cryoprotectant. X-ray diffraction data were collected at the X06SA beamline at the Swiss Light Source (Villigen, Switzerland). Data processing and initial phase determination were carried out in the same manner as previously described [27]. The generation of the topology and parameter file for the compound, and structure refinement, were performed as described previously [25]. The structural coordinates and structure factors have been deposited in the Protein Data Bank with the accession code 8INL.

### Enzyme inhibition assay

Enzyme inhibition assays against LSD1, LSD2, MAO-A, and MAO-B were performed as previously described [25]. Technical replicates, *n* = 3.

### Pharmacokinetic analysis

Male Crl:CD1 (ICR) mice from Jackson Laboratories, Japan (8 weeks old, *n* = 3) received a single intraperitoneal injection of S2172 at 30 mg/kg in 5 mL/kg of 15% DMSO/17.5% Cremophor EL/8.75% Ethanol/8.75% HCO-40/50% PBS. Serial blood sampling was conducted from the jugular vein at 0.25, 0.5, 1, 2, 4, and 6 hr time points. At 0.5 h after injection, mice were euthanized, underwent hemoperfusion, and their brain tissue was harvested, homogenized and diluted 4-fold with water. Blood and brain homogenate samples were treated with acetonitrile and centrifuged. The supernatant solution was used for drug level measurement by LC/MS/MS. Pharmacokinetic parameters were calculated using Moment method analysis.

### Chromatin immune precipitation (ChIP)

Cells (1 × 10^6^) were treated with 1% formaldehyde for 8 min to crosslink histones to DNA. After washing with cold PBS, cell pellets were lysed with lysis buffer (10 mM Tris-HCl, 0.25% Triton X-100, 10 mM EDTA, and 0.1 M NaCl) [28]. Cell nuclei were collected after centrifugation and re-suspended in SDS lysis buffer (1% SDS, 150 mM NaCl, 50 mM HEPES-KOH, 2 mM EDTA, 1% Triton X-100, and 0.1% sodium deoxycholate). Chromatin was sonicated using Covaris S220 (Covaris, Moburn, MA, USA). Lysates were incubated overnight with 50 μL of Dynabeads Protein G (Thermo Fisher Scientific) which were pre-coated with 4 μL of anti-H3K4me1 antibody (ab8895, Abcam, Cambridge, UK), 3 μL of anti-H3K4me2 antibody (#39141, Active Motif), 2.5 μL of anti-H3K4me3 antibody (#39159, Active Motif), 4 μL of anti-H3K9me2 antibody (ab1220, Abcam). The beads were washed and protein-DNA complexes were eluted with elution buffer (1% SDS, 10 mM EDTA, and 50 mM Tris-HCl) and then treated with RNase for 2 h at 37°C followed by proteinase K treatment overnight. DNA was extracted by the phenol/chloroform method, ethanol-precipitated and re-suspended in 0.1 × TE buffer. 10% of the lysate was treated with RNase and proteinase K and de-crosslinked as input samples.

ChIP DNA (*n* = 2 for each antibody in each condition) was used to make sequence libraries using NEBNext Ultra II DNA library Prep kit for the Illumina (New England Biolabs, Ipswich, MA, USA). Library quality was checked using the Agilent Bioanalyzer 2100 (Agilent Technologies, Santa Clara, CA, USA) and then libraries were run on an Illumina Hiseq XTen (Illumina, San Diego, CA, USA).

### ChIP-seq data analysis and identification of super-enhancer

ChIP-seq clean reads were obtained from raw reads by the removal of adaptors and low-quality reads. The clean reads were aligned to the human genome (hg38) using Bowtie2 (version 2.4.2) [29]. The levels of histone modification at gene promoters were estimated by counting the numbers of reads mapped within a 2-kb region around the transcriptional start site using MACS2 [30] and HOMER [31]. Differential analysis of peaks and generation of heatmap were performed using BEDtools v2.30.0 [32]. HOMER was used to annotate ChIP-seq peaks for genomic location and link these peaks to nearby genes. TF binding motif analysis was performed using HOMER. BEDtools was used to obtain overlap peaks with super-enhancers and H3K4me2 peaks. Furthermore, we used our deposited GSC316 H3K27ac ChIP-seq data (GSE178471) [33] and identified super-enhancers by the Rank Ordering of Super Enhancer (ROSE) algorithm [20, 34]. From this analysis, we identified 672 super-enhancers.

### RNA-seq

GSC cells treated with either DMSO (*n* = 4) or S2172 (5 μM, *n* = 4) for 96 h and total RNA was isolated with RNeasy mini kit (Qiagen). The RNA purity was determined using Agilent Tape Station (Agilent Technologies). Illumina TruSeq RNA sample preparation was conducted and the samples were run on an Illumina Hiseq XTen (Illumina). RNA-seq was aligned using HISAT2 under default setting to Homo Sapience hg38 [35]. TPM were generated using StringTie [36] and differential expression analysis were performed using DESeq2 [37].

### Animal experiments

All experiments were performed under protocols approved by the Institutional Animal Care and Use Committee of Nagoya University of Graduate School of Medicine. Cells (1 × 10^5^ cells per mouse) were injected intracranially into 6-week-old female NOD/SCID mice (*n* = 20, SLC, Shizuoka, Japan). Four weeks after the injection, S2172 or DMSO were intravenously injected five days a week for 3 weeks (10 mg/kg per day, 15 injections in total). We used established guidelines to determine the sample size for the mouse experiments [38, 39]. The mice were assigned randomly to experimental and control groups. There was no blinding applied in the experiment.

### Statistics

Data in bar graphs represent the mean ± SD of at least three biological repeats. Statistical analysis was performed using a two-sided Student’s t-test by comparing chemical versus DMSO treatment. Statistical analysis and visualization were performed with GraphPad Prism 7 (GraphPad Software, San Diego, CA, USA) and R (version 4.2.2). The center values represent the mean. All reported *P* values are two-sided, with *P* < 0.05 taken as statistically significant. Variance within each group was estimated by calculating standard deviation, and similar variances between groups were confirmed prior to statistical analysis.

## Results

### Development of LSD1 inhibitor, S2172

**Fig. 1:**
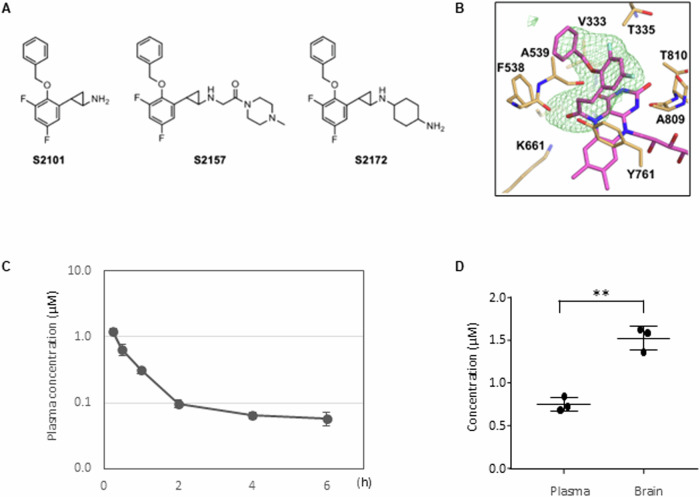
Development of LSD1 inhibitor, S2172. **A** Chemical structures of S2101, S2157, and S2172. **B** Crystal structure of S2172 bound to LSD1. The adduct of S2172−FAD and nearby residues of LSD1 are colored magenta and orange, respectively. The mFo−DFc electron density map at +3.0 σ, calculated without the inhibitor portion of the adduct, is shown as a green mesh. **C** Kinetics of S2172 in plasma after injection of 30 mg/kg of S2172 in ICR mice (*n* = 3). **D** Concentration of S2172 30 min after injection of 30 mg/kg of S2172 in ICR mice (*n* = 3). The *y* axis indicates concentration of S2172 (μM). Error bars indicate the SD. **P < 0.01.

We previously developed an *N*-alkylated *trans*-2-phenylcyclopropylamine (2-PCPA)-based inhibitor, S2157 [25], in reference to the 2-PCPA-based cell-permeable LSD1 inhibitor, S2101 [23] (Fig. 1A). In this study, we downsized the *N*-alkyl group of S2157 to synthesize a compound named S2172 and investigated its efficacy as an LSD1 inhibitor. First, we measured the inhibitory activity of S2172 against LSD1 and other FAD-dependent enzymes, such as LSD2, monoamine oxidase A (MAO-A), and MAO-B, in an in vitro peroxidase assay (Table 1). S2172 exhibited stronger LSD1 inhibitory activity than S2157 (*K*_i_ value of 0.27 ± 0.06 vs. 0.37 ± 0.08 µM), while the other three enzymes were not inhibited. S2172 had a *k*_inact_/*K*_i_ value of 9800 ± 570 M^−1^ s^−1^ (Supplementary Table S2), which was better than the reported value of 7800 M^−1^ s^−1^ for S2157 [25].

**Table 1:** Effects of 2-PCPA-based cell-permeable LSD1 inhibitors against FAD-dependent enzymes.

Compound	In vitro *i*nhibition parameters *Ki* (µM)			
	LSD1	LSD2	MAO-A	MAO-B
2-PCPA*	100 ± 22 [23]	>250 [25]	5.0 ± 0.70 [23]	26 ± 0.082 [23]
S2101*	0.61 ± 0.13 [23]	>250 [25]	110 ± 11 [23]	17 ± 2.5 [23]
S2157*	0.37 ± 0.08 [25]	>250 [25]	>250 [25]	>250 [25]
S2172	0.27 ± 0.06	>250	>250	>250

^*^, Values reported in Mimasu et al. [23] and Niwa et al. [25] are indicated by literature number. *2-PCPA* *trans*-2-phenylcyclopropylamine (also known as tranylcypromine). Technical replicates, *n* = 3.

To analyze the mechanism of inhibition, we next solved the crystal structure of LSD1 in complex with S2172. Crystallographic statistics are summarized in Table 2. In the crystal resolved at 2.62 Å, S2172 formed a covalently linked adduct with FAD in the catalytic center of LSD1, through a cyclopropyl carbon atom (Fig. 1B). The overall structure of the LSD1–S2172 adduct was similar to that of LSD1–S2101 or LSD1–S2157 [25], suggesting that S2172 can inactivate LSD1 by a mechanism similar to those inhibitors.

**Table 2:** Crystallographic statistics for LSD1−S2172 complex.

PDB ID	8INL
**Data collection and processing**	
Beamline	SLS X06SA
Wavelength (Å)	1.000
Space group	*P*6_1_22
Unit cell dimensions	
* a*, *b*, *c* (Å)	187.1, 187.1, 106.9
* α, β, γ* (°)	90, 90, 120
Resolution (Å)	48.21−2.62 (2.74−2.62)^*^
No. of unique reflections	33,642 (4040)
Completeness (%)	100 (100)
Multiplicity	40.9 (42.8)
Mean *I*/*σ(I)*	22.3 (1.8)
*R*_sym_	0.136 (3.725)
*R*_pim_	0.021 (0.574)
CC_1/2_	1.000 (0.798)
**Refinement**	
*R*_work_/*R*_free_	0.180/0.216
R.m.s.d. from ideal	
Bond lengths (Å)	0.006
Bond angles (°)	0.801
No. of atoms	
Total	5251
Protein/adduct/water/others	5081/73/57/40
Mean B-factor (Å^2^)	
Overall	86.9
Protein/adduct/water/others	87.3/67.2/72.8/88.8
Ramachandran plot (%)	
Favored	97.0
Allowed	3.0
Outliers	0.0

^*^, Values in parenthesis are for the highest resolution bin.

Our prior LSD1 inhibitor, S2157, effectively eliminated leukemia cells in the central nervous system of mice transplanted with T-cell acute lymphoblastic leukemia cells, as it successfully crossed the blood-brain barrier [26]. Consequently, we examined the pharmacokinetics of S2172 in plasma and its penetration into the brain in mice (Fig. 1C, D). Thirty minutes after a single injection of 30 mg/kg via the intraperitoneal route, S2172 reached a brain concentration of 1.52 ± 0.14 µM and a maximum plasma concentration of 0.75 ± 0.09 µM at 0.5 h, indicating a good brain-to-plasma concentration ratio (2.06 ± 0.39) [40] (Fig. 1D, Table 3). Therefore, we proceeded to investigate the anticancer effects of S2172 against glioma cells, particularly GSCs, through in vitro and in vivo experiments.

**Table 3:** Pharmacokinetic parameters of S2172.

C_max_ (µM)	1.20 ± 0.12
T_max_ (hr)	0.25
T_1/2_ (hr)	4.98 ± 1.64
AUC_0–6 h_ (µM·hr)	1.11 ± 0.08
Plasma concentration at 0.5 hr (µM)	0.75 ± 0.09
Brain concentration at 0.5 hr (µM)	1.52 ± 0.14
Brain/plasma ratio	2.06 ± 0.39

Pharmacokinetic parameters were determined in 8-week-old Crl:CD1 (ICR) mice treated with a single intraperitoneal injection at 30 mg/kg (*n* = 3).

### S2172 has an antitumor effect on glioma cells

We examined LSD1 expression levels in clinical glioma samples using data from The Cancer Genome Atlas (TCGA) database. Analysis of TCGA data revealed significantly higher LSD1 expression levels in gliomas (*n* = 157) compared to normal counterparts (*n* = 5) (*P* < 0.01, Supplementary Fig. S1B). LSD1 expression was detected in three glioma stem cell (GSC) lines (GSC1228, GSC222, and GSC316), as well as in differentiated glioma cell lines (LN229), and a normal neural stem cell line (F3), showing substantial but variable levels of both mRNA and protein expression (Supplementary Fig. S1C, D).

Inhibition of LSD1 can effectively suppress stem-like tumor-propagating cells in human glioma [24]. Therefore, we treated glioma stem cell lines, GSC1228, GSC222, and GSC316, which express stemness markers (MYC, Nestin, and SOX2)(Supplementary Fig. S1C, D) and possess the potential for self-renewal, differentiation, and tumor-initiating capacity [7, 41], with S2101, S2157, and S2172 for 96 h and evaluated the growth inhibitory effect. S2172 exhibited a stronger inhibitory effect on GSC1228 cell lines compared to S2101 and S2157 (Supplementary Fig. S1E, *P* = 0.0077, <0.0001, <0.0001, at 10 μM, 20 μM, and 40 μM, respectively). The growth inhibitory IC50 (GI50) of S2172 in all three GSCs was smaller than that of S2101 and S2157 (12.5, 17, and 12 μM in GSC1228, GSC222, GSC316, respectively; Table 4). In differentiated glioma cell lines (LN229), we observed a similar trend, with lower doses of S2172 inhibiting cell growth more effectively (GI50: 8.2 μM) compared to S2101 and S2157 (Table 4). Grade III glioma cell lines showed slightly higher GI50 (33 μM, 21 μM, 17 μM in KINGS1, Onda10, and TM31, respectively) compare to those of GSCs and LN229 (GBM cell line) (Table 4). While the GI50 of S2172 in the normal neural stem cell line F3 was substantially higher than in other glioma cell lines, F3 was more sensitive to S2172 than the normal fibroblast cell lines TIG3 and WI38 (22.5 μM, >40 μM, and 29.9 μM in F3, TIG3, and WI38, respectively; Table 4).

**Table 4:** Growth inhibitory IC50 of S2101, S2157, and S2172 in cancer cell lines.

	S2101 (µM)	S2157 (µM)	S2172 (µM)
GSC1228	>40	19.7	12.5
GSC222	>40	>40	17.0
GSC316	>40	>40	12.0
LN229	12.0	>40	8.2
KINGS1	19.0	>40	33
Onda10	25.0	>40	21
TM31	6.9	27	17
F3	26.0	36.5	22.5
TIG3	>40	33.3	>40
WI38	>40	>40	29.9

### Effects of S2172 on histone modifications in GSCs

**Fig. 2:**
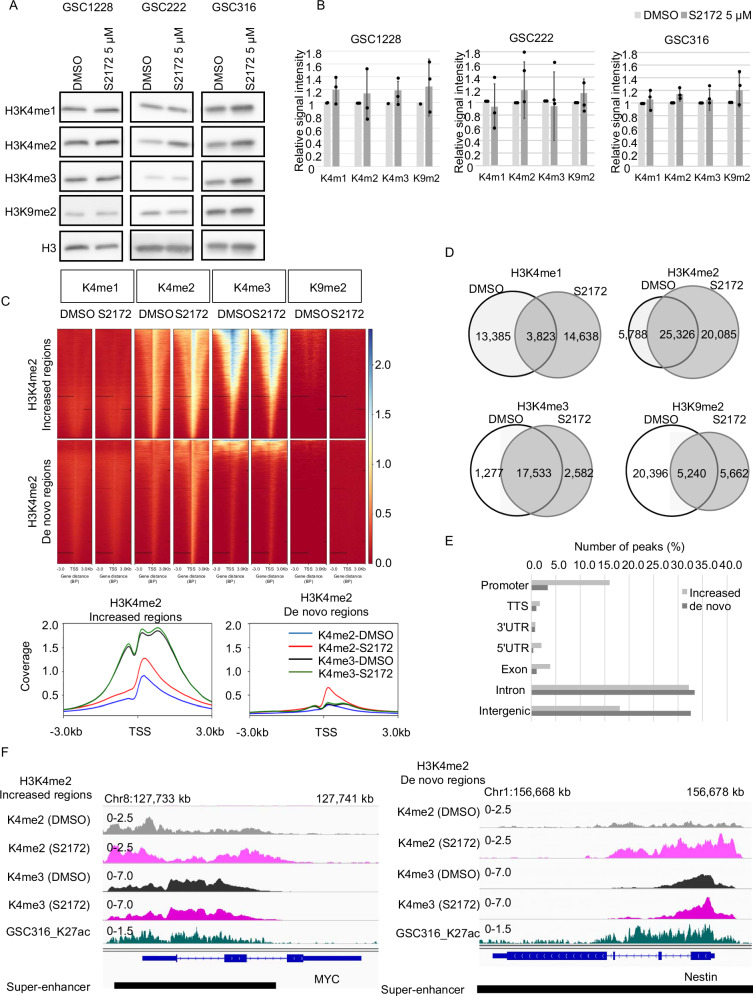
S2172 affected H3K4me2 modifications. **A** Western blot analysis of H3K4me1, H3K4me2, H3K4me3, H3K9me2, and H3 after treatment with either DMSO or S2172 (5 μM) for 96 h in GSC1228, GSC222, and GSC316. Histone H3 was used as the loading control. **B** Quantification of band signal intensities from the western blot of **A**. The y-axis in the panel indicates relative signal intensity of each protein normalized to histone H3. Error bars indicate the SD. **C** Upper panel, ChIP-seq peaks (3 kb upstream and downstream of the H3K4me2-binding site) of H3K4me1, H3K4me2, H3K4me3 and H3K9me2. Signal intensity is shown on the right. Lower panel, Distribution of H3K4me2 and H3K4me3 signals at H3K4me2-binding site in GSC1228 treated with DMSO or S2172 (5 μM). **D** Peaks of H3K4me1, H3K4me2, H3K4me3 were identified using HOMER findPeaks function with histone setting. Venn diagrams for each binding peak are shown. **E** Distribution of H3K4me2 peaks at the ‘increased’ and ‘de novo’ regions. **F** Representative ChIP-seq peaks at the MYC and Nestin loci.

The effects of S2172 on global histone modifications in GSCs were examined using western blot analysis. GSC cells were treated with 5 μM of S2172 since <80% of the cells are still viable at this concentration (Fig. 2, Supplementary Fig. S1E). This 5 μM treatment resulted in greater changes in histone modifications compared to those treated with 1 or 10 μM of S2172 (Supplementary Fig. S1F, G). Treatment of GSCs with S2172 increased the overall levels of H3K4me2 as well as H3K9me2. However, these changes were not statistically significant (Fig. 2A, B). Interestingly, the level of H3K4me2 was significantly increased in LN229 after treatment with S2172 (Supplementary Fig. S2A). Among grade III glioma cell lines, KINGS1 was treated with S2172 because it showed a similar expression level of LSD1 as GSC1228 (Supplementary Fig. S3A, B). After treatment with S2172, there was a slight increase in the levels of H3K4me1, H3K4me2, H3K4me3, and H3K9me2 (Supplementary Fig. S3C). The neural stem cell line F3 was also treated with S2172, but the changes in the levels of H3K4me1, H3K4me2, H3K4me3, and H3K9me2 were not significant (Supplementary Fig. S4A).

To precisely evaluate the effects of S2172 on histone modifications at a genome-wide scale, we performed ChIP-seq analysis in GSC1228 cells treated with either DMSO or S2172. Since GSC1228 was comprehensively studied in our previous research, demonstrating stable proliferation and the ability of establish brain tumors in mice [[7, 41], Katsushima, 2012 #1010], we chose this cell line for Chip-seq analysis. S2172 treatment clearly altered the profile of H3K4me1, -me2, -me3, and H3K9me2 marks (Fig. 2C). These alterations were particularly obvious for H3K4me2, which appeared 20,085 new regions (de novo regions), but was lost from 5788 regions (Fig. 2D). Notably, 25,326 regions already modified by H3K4me2 before the treatment showed increased levels of H3K4me2 after S2172 treatment (Fig. 2C). The increased in H3K4me2 after S2172 treatment was enriched at promoter regions, intronic and intergenic regions (Fig. 2E). In contrast to other modifications, much fewer de novo H3K4me3 regions (2582 regions) were observed after treatment; this is consistent with S2172 inhibiting LSD1, which catalyzes H3K4me1/me2 demethylation (Fig. 2C, D).

**Fig. 3:**
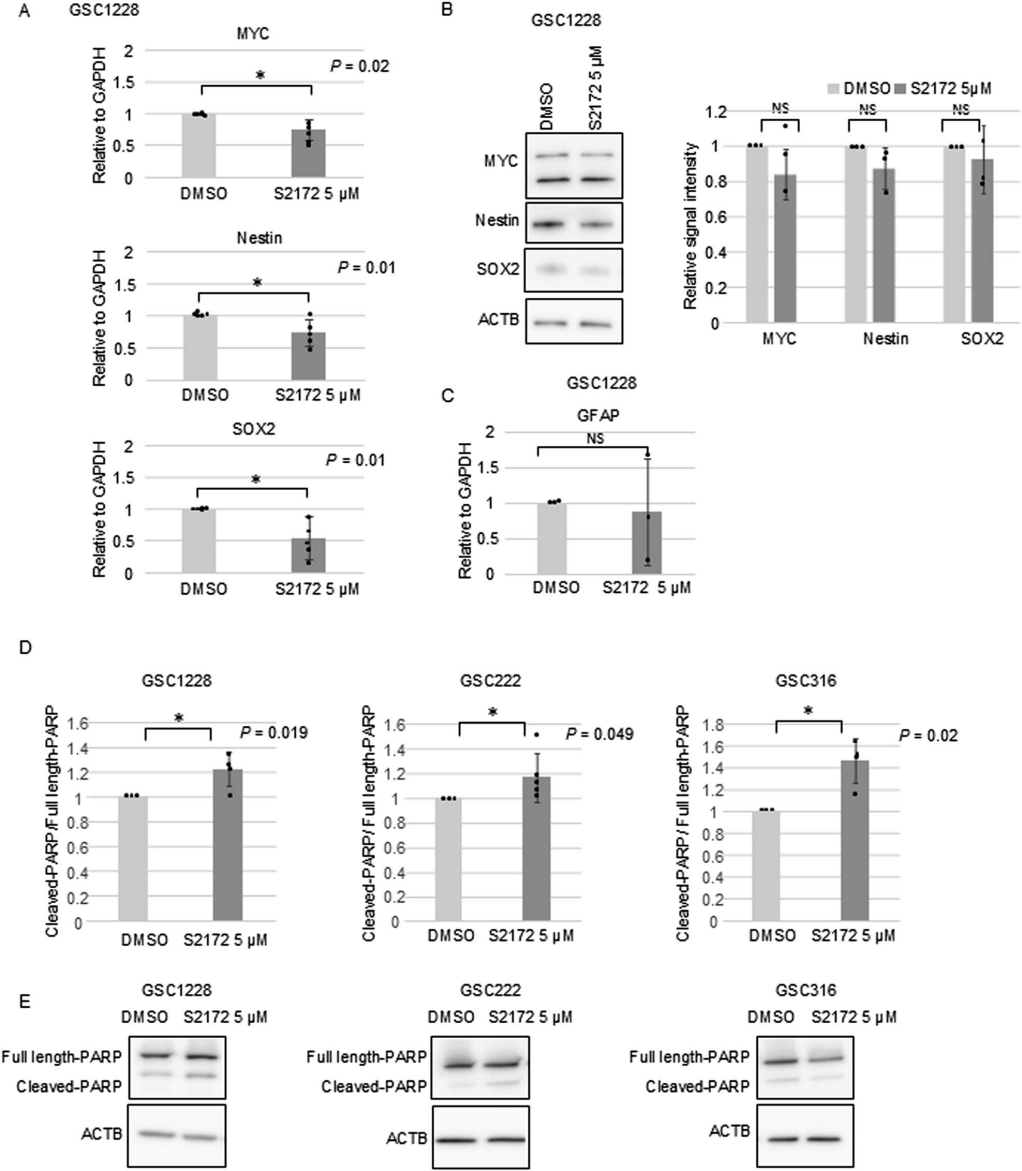
S2172 treatment downregulated the stemness-associated genes in GSC1228. **A** mRNA expression of stem cell marker genes following 96 h treatment of GSC1228 with S2172 (5 μM). The y-axis indicates the expression change relative to DMSO-treated cells. Error bar indicates the SD. *P < 0.05. **B** Left panel, western blot analysis of MYC, Nestin, and SOX2 after treatment of GSC1228 with either DMSO or S2172 (5 μM) for 96 h. β-actin (ACTB) was used as the loading control. Right panel, Quantification of band signal intensities from the western blot. **C** mRNA expression of the differentiation marker gene, GFAP, following treatment of GSC1228 with S2172 (5 μM) for 96 h. The y-axis indicates the expression change relative to DMSO-treated cells. Error bar indicates the SD. **D** PARP cleavage ratio after treatment of GSC1228, GSC222, and GSC316 with S2172 (5 μM). The ratio was calculated by dividing the cleaved-PARP band intensity by the full length-PARP band intensity. Error bar indicates the SD. *P < 0.05. **E** Representative Western blot results of RAPR after treatment of GSC1228, GSC222, and GSC316 with either DMSO or S2172 (5 μM) for 96 h. The full length-PARP band (106 kDa) and cleaved PARP band (89 kDa) are shown. β-actin (ACTB) was used as the loading control. *n* = 4.

Super-enhancers consist of enhancer clusters with abundant binding of transcription factors and are highly enriched with H3K27ac marks [20, 34]. The H3K4me2 increased regions included the MYC locus and H3K4me2 de novo regions comprised Nestin and SOX2 loci (Fig. 2F, Supplementary Fig. S5A). These regions were also identified as super-enhancers in GSCs using data from our previous study [33] (Fig. 2F). Compared to the large increase in H3K4me2 in these regions, changes in H3K4me3 were subtle after S2172 treatment (only 2582 regions were acquired modification) (Fig. 2C). Increasing the me2/me3 ratio at H3K4 is associated with reduced gene expression [42]. Indeed, these three stemness marker genes were significantly downregulated at the transcriptional level (mRNA) and tended to be downregulated at the protein level in GSC1228 following treatment with S2172 (Fig. 3A, B). While another LSD1 inhibitor was reported to induce differentiation of GSC into astrocyte [43], S2172 did not transcriptionally induce the astrocytic marker, GFAP, in this experimental setting (Fig. 3C). Treatment with S2172 for 24 h induced apoptosis in GSC and LN229 cells as evidenced by increased cleaved poly (ADP-ribose) polymerase (PARP) (*P* = 0.019, 0. 049, 0.02, and 0.03 in GSC1228, GSC222, GSC316, and LN229, respectively) (Fig. 3D, Supplementary Fig. S2B). Contrary to the GSC cells, KINGS1 did not show significant changes in stemness marker expression at either mRNA or protein levels (Supplementary Fig. S3D, E). In F3 cell, the expression levels of stemness markers were not significantly affected (Supplementary Fig. S4B, C). Cleaved-PARP levels in KINGS1 and F3 cells were not significantly increased by this concentration of S2172 treatment (Supplementary Figs. S3D, F and S4D). Taken together, these data suggest that S2172 exerts a more potent effect on GBM, particularly on GSC populations.

### S2172 affects super-enhancer-dependent regulation of gene expression in GSCs

As mentioned earlier, an increase in H3K4me2 was observed after S2172 treatment within super-enhancer regions of MYC, Nestin, and SOX2 loci (Fig. 2F, Supplementary Fig. S5A). We further investigated whether the modifications within enhancers/super-enhancers were globally affected by the S2172 treatment. We identified 672 super-enhancers and 11,043 typical enhancers in GSCs by H3K27ac signal ranking [33, 44] (Materials and Methods, Supplementary Fig S6A). Of the 20,085 de novo H3K4me2 peaks, 284 (1.4%) overlapped with super-enhancers, and 2386 (11.9%) overlapped with typical enhancers. Among the 25,326 increased H3K4me2 peaks, 911 (3.6%) overlapped with super-enhancers, and 5943 (23.5%) were within typical enhancers. Motif enrichment analysis revealed that binding motif of transcription factors IRF1, Atf3, and DLX5 were enriched in de novo H3K4me2 regions (Supplementary Fig. S5B).

Next, we investigated whether changes in H3K4me2 within enhancers had an impact on RNA expression levels. RNA-seq analysis was conducted to examine gene expression changes associated with super-enhancers/enhancers. Among the genes with de novo H3K4me2 modification, the average log_2_ fold changes (S2172 treatment relative to DMSO treatment) were −0.12 ± 0.03 for super-enhancer-related genes and −0.01 ± 0.01 for typical enhancer-related genes (*P* = 0.0004) (Supplementary Fig. S6B). In contrast, among the genes with increased H3K4me2 modification, the average log_2_ fold changes were −0.04 ± 0.01 for super-enhancer-related genes and −0.07 ± 001 for typical enhancer-related genes (*P* = 0.028) (Supplementary Fig. S6C). Thus, super-enhancer-regulated genes among de novo H3K4me2-modified genes were significantly downregulated, while typical enhancer-related genes were more affected by increased H3K4me2 modification after S2172 treatment. In addition to *MYC*, *Nestin*, and *SOX2* (Fig. 2F, Supplementary Fig. S5A), we further found that super-enhancer-associated genes, early growth response 1 (*EGR1*), and cyclin-dependent kinase 6 (*CDK6*) (Supplementary Fig. S7A) were downregulated concomitant with an increase in H3K4me2 upon S2172 treatment in GSC cells (Supplementary Fig. S7A, B).

Super-enhancers are regions characterized by binding of abundant transcription factors and mediators, leading to strong transcription activity. Among these factors, BRD4, a member of bromodomain and extraterminal (BET) protein family, plays a crucial role in regulating super-enhancer-associated genes. We treated GSC1228 cells with S2172 in combination with the BRD4 inhibitor JQ-1. As shown in Fig. 3A, B, S2172 significantly downregulated MYC expression at the mRNA level, but not at the protein level. However, the combination treatment of S2172 and JQ-1 resulted in a significant suppression of MYC protein expression (*P* < 0.05) (Supplementary Fig. S8A, B).

### S2172 treatment in vivo

Finally, we examined the therapeutic effects of S2172 in vivo. To determine the tolerated dose of S2172 in mice, we administered doses of 10 mg/kg and 50 mg/kg. Since previously developed LSD1 inhibitor S2157 was administered at a dose of 50 mg/kg [26] we used this concentration as a reference. During the treatment period, mice treated with 10 mg/kg of S2172 exhibited no obvious physical changes, while severe body weight loss was observed in mice treated with 50 mg/kg (Supplementary Fig. S9A). In addition, three mice died during the treatment with 50 mg/kg of S2172. Therefore, we used 10 mg/kg of S2172 for further in vivo analysis.

**Fig. 4:**
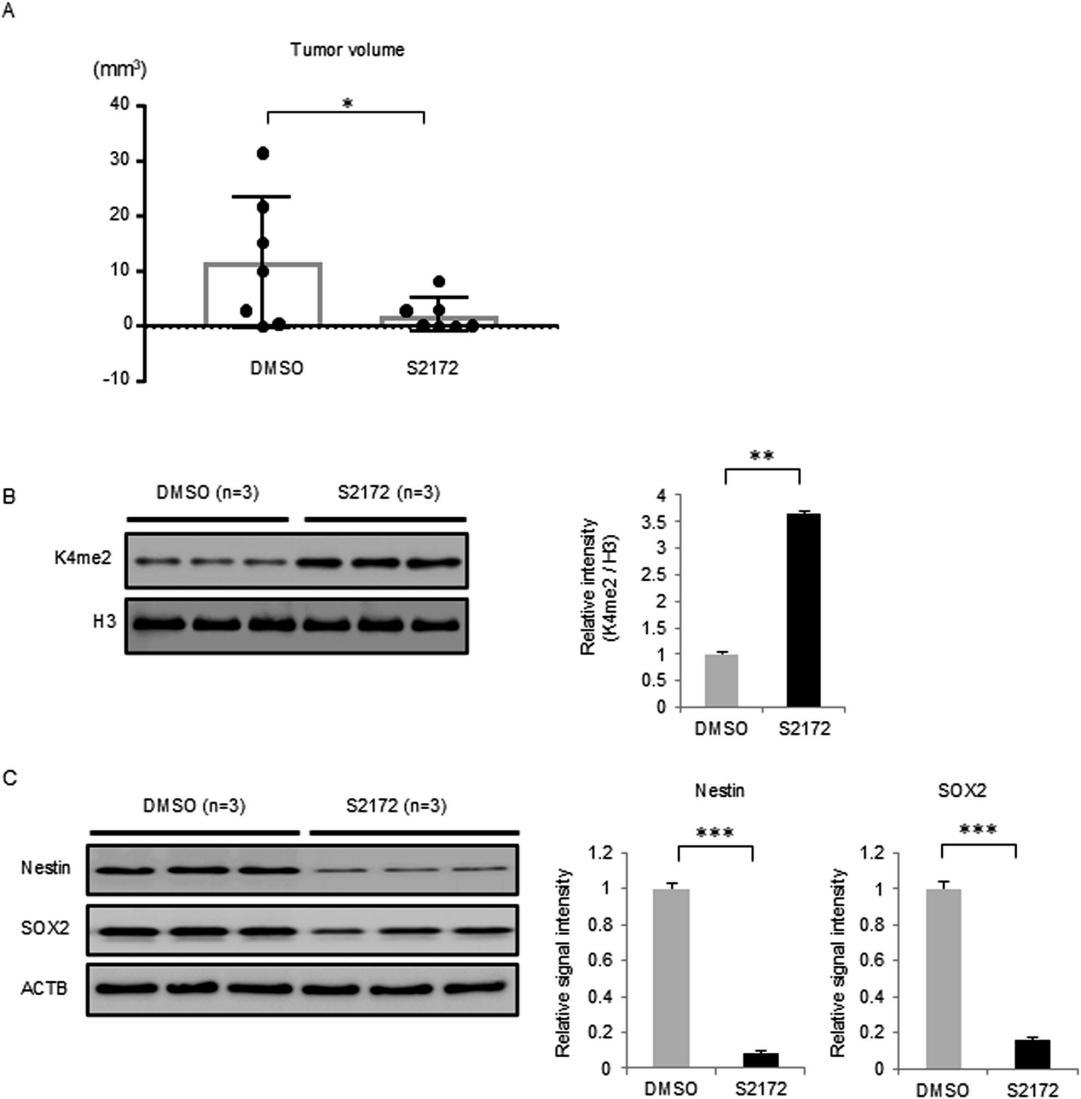
S2172 eradicates tumor cells in GSC brain tumor mouse model. **A** Tumor volume was assessed by MRI after treatment with either DMSO or S2172 for 15 times. Error bars represent the SD. *P < 0.05. *n* = 7. **B** H3K4me2 level in the tumor tissue from the mouse brain (left panel). Quantification of western blot band signal intensity (right panel). The y-axis in the panel indicates the relative signal intensity of each protein normalized to histone H3. Error bars indicate the SD. **P < 0.01. **C** Level of Nestin and SOX2 proteins in the tumor tissue from mouse brains after treatment with DMSO or S2172 (Left panel). Quantification of western blot band signal intensity (right panel). The y-axis in the panel indicates the relative signal intensity of each protein normalized to β-actin (ACTB). Error bars indicate the SD. ***P < 0.001.

We utilized intracranial xenograft mouse models inoculated with GSC1228 cells. After 30 days of inoculation, we administered S2172 intravenously at 10 mg/kg five times a week for three weeks. The tumor area was significantly smaller in mice treated with S2172 (*P* < 0.05, Fig. 4A, as assessed by MRI; in an additional set of experiments, *P* < 0.01, as assessed by HE-stained tissue images, Supplementary Fig. S9B). Following treatment with S2172 for one week, mice were sacrificed, and the xenograft glioma tissues were examined. The H3K4me2 level was increased in the tumor regions treated with S2172 (*P* < 0.01, Fig. 4B). Consistent with the in vitro analysis, the protein levels of Nestin and SOX2 were decreased in tumor tissues (Fig. 4C).

## Discussion

Given the promise of LSD1 for the therapeutic targeting of glioma stem cells [24, 43], we aimed to develop a more potent LSD1 inhibitor with brain tissue penetration. S2172 inhibited the growth of GSCs both in vitro and in vivo by diminishing their stemness, leading to apoptosis. This is consistent with previous reports demonstrating that LSD1 inhibition is a potent suppressor of GSC growth [24, 43].

Previous chromatin mapping of the genome suggests that elevated ratios of H3K4me2/me3 are detected at poised or weakly active promoters compared to fully activated promoters [42, 45]. Indeed, in MLL-AF9 leukemia cells treated with an LSD1 inhibitor, an increased H3K4me2/me3 ratio at MLL-AF9-bound genes was correlated with decreased expression. Continued expression of MLL-AF9-bound genes may be directly dependent upon the demethylase activity of LSD1 settled in the region [42]. After treatment of GSC1228 with S2172, we observed genome-wide enrichment of H3K4me2 at certain loci, such as enhancers/super-enhancers, but not significant changes in H3K4me3 modification. The net effect of this is likely an increased ratio of H3K4me2/me3 and a decrease in transcription, including the stemness-related genes *MYC*, *SOX2*, and *Nestin*.

Super-enhancers are clusters of enhancers densely populated by transcriptional coactivators and marked by active chromatin signatures such as H3K27ac; they orchestrate the expression of genes that play pivotal roles in cell-type-specific processes [20, 34, 46]. The acquisition of super-enhancers that regulate the expression of genes involved in cancer initiation and progression contributes to tumorigenesis [34, 47] Super-enhancers have been found near the MYC gene, resulting in altered chromatin structures and high production of MYC mRNA and protein. The activity of these super-enhancers is perturbed by epigenetic treatment, leading to the downregulation of MYC [34]. In addition, LSD1 interacts with BRD4 and colocalizes with this epigenetic regulator at super-enhancers in breast cancer and prostate cancer [48, 49]. In our study, LSD1 inhibition led to the downregulation of *MYC* via dynamic changes in H3K4 methylation at the *MYC* locus. Furthermore, the combination treatment with a BRD4 inhibitor efficiently suppressed MYC protein expression. Stemness-related genes, such as *SOX2* and *POUF2*, are essential for glioma propagation [24], and MYC regulates the expression of these genes in glioma cells [21]. Notably, motif analysis of H3K4me2 peaks revealed an enrichment of Atf3 in de novo H3K4me2 peaks, where the Nestin and SOX2 loci were involved. Since LSD1 and MYC co-localize at Atf3 sites [50], we infer that LSD1 inhibition might induce changes in H3K4me2, leading to the remodeling of chromatin structure within super-enhancers, particularly at the SOX2 and Nestin loci. Consequently, expression of these genes is altered by the LSD1 inhibitor, S2172.

In addition to stemness-associated genes regulated by super-enhancers in de novo H3K4me2-modified genes, we found that *EGR1* and *CDK6* were also consistently downregulated by S2172 treatment in GSC cells. EGR1 is a zinc finger transcription factor and an early response gene that is rapidly induced by environmental stimuli [51]. EGR1 binds to the cyclin D2 promoter and regulates its expression in prostate cancer cell lines [52]. EGR1 overexpressed in several cancers types, consistent with its known oncogenic role [53, 54]. In addition, EGR1 plays an important role in the maintenance of stemness marker expression, depending on platelet-derived growth factor subunit A (*PDGFA*) expression [55]. CDK6, a member of the cyclin-dependent kinase family, encodes a protein involved in cell cycle regulation. The association of CDK4 or CDK6 with D-type cyclins is essential for G1 phase progression [56]. Pharmacological inhibitors targeting CDK4/6 have been developed based on the rationale that inhibiting CDK activity will block cell proliferation, thereby mitigating cancer pathogenesis [57]. Besides these genes, ontology analysis by GREAT [58] identified many differentiation-related pathways in super-enhancer-regulated genes within de novo H3K4me2-modified peaks. In addition to the effect to cancer cells, LSD1 inhibitors have been reported to enhance NK cell cytotoxicity or T-cell infiltration in vivo [59, 60]. The impact of S2172 in the mouse model may be due to the involvement of the immune system, contributing to the significant in vivo response observed. The dysregulation of these gene regulatory networks following S2172 treatment is expected to be elucidated in the future and could potentially be exploited therapeutically to target and eradicate GSCs (Supplementary Figure. S10).

In summary, we identified a specific and potent LSD1 inhibitor that successfully crossed the blood-brain barrier following intravenous injection. Through the disruption of super-enhancer activity, inhibiting LSD1 with S2172 blocked stemness and reduced the viability of GSC cells. Thus, targeting LSD1 with our novel compound, S2172, could be a potent therapeutic approach for malignant glioma, a tumor characterized by an abundance of GSCs. Further clinical research is needed to validate this hypothesis.

## Supplementary information

Supplementary items

## Data Availability

Datasets related to this article have been submitted to an open-source data repository, Gene Expression Omnibus (GEO, https://www.ncbi.nlm.nih.gov/geo/). Accession numbers are GSE261471 and GSE261472.
