# Supplementary material for: Novel pharmacologic inhibition of lysine-specific demethylase 1 as a potential therapeutic for glioblastoma
Source: Cancer Gene Ther. 2024 Nov 5;31(12):1884–94. doi: 10.1038/s41417-024-00847-8 (PMC11645267; doi:10.1038/s41417-024-00847-8)
Supplement: Supplementary file 1 — Supplementary items [file 41417_2024_847_MOESM1_ESM.pdf]

Supplementary Items

1. Supplementary materials and methods

2. Supplementary Figures S1 to S6

3. Supplementary Tables S1 to S2

Supplementary materials and methods

TCGA data

RNA-seq data of malignant glioma (n=157) and normal tissue (n=5) were obtained from TCGA (http://xena.ucsc.edu/).

Cell lines and culture conditions

Glioblastoma cell line, LN229, was obtained from the American Type Culture Collection (ATCC, Manassas, VA, USA). Normal fibroblast cell lines TIG3 and WI-38, TM31 (low grade glioma) were obtained from the RIKEN Bioresource Research Center (Tsukuba, Japan). Onda10 and KINGS1 (low grade glioma) cell lines were obtained from the Japanese Collection of Research Bioresources (Ibaraki, Japan). F3 is utilized as a neural stem cell line{Kim, 2004 #1412}{Natsume, 2013 #1009}. All cells were maintained in DMEM medium (Wako, Osaka, Japan) containing 5% fetal bovine serum (FBS, Thermo Fisher Scientific, Waltham, MA, USA) and 1% penicillin-streptomycin (Wako). Glioma stem cell lines (GSC1228, GSC316, and GSC222) were established previously{Natsume, 2013 #1009}{Katsushima, 2016 #1156}. GSC cells were cultured in Neurobasal Medium (Life Technologies, Carlsbad, CA, USA) containing N2 and B27 supplements (Life Technologies) along with human recombinant basic fibroblast growth factor and epidermal growth factor (20 ng/ml each; R&D Systems, Minneapolis, MN, USA). Cells were cultured at 37°C in a humidified incubator with 5% CO_2.

For the cell proliferation assay, cells at a density of 1×10^5 cells per well were seeded before treatment with LSD1 inhibitors. Cell proliferation was measured after 4 days using a Cell Counting Kit 8 (Dojindo, Kumamoto, Japan) according to the manufacturer's

instructions. For GSC cells, the CellTiter-Glo 3D Viability Assay (Promega, Madison, WI, USA) was used to measure the living cells. All proliferation experiments were repeated more than three times.

RNA extraction

Total RNA from the cell lines was extracted using TRIzol (Thermo Fisher Scientific), followed by reverse-transcription using Prime Script RT Master Mix (Takara, Kusatsu, Japan). TaqMan qPCR (Roche Diagnostics, Basel, Switzerland) and SYBR Green qPCR (TOYOBO, Osaka, Japan) were performed at least in triplicate for the target genes. Expression levels of each gene were normalized to GAPDH. GAPDH for TaqMan PCR assays was Hs.PT.39a.22214836 (Integrated DNA Technologies, Coralville, IA, USA). Oligonucleotide primers used for SYBR Green assays are shown in Supplementary Table S1.

Western blot analysis

The cell lysates were extracted from treated cells. A total of 100 µg of protein was separated on 10% SDS/PAGE gels, transferred to nitrocellulose membranes and incubated with the following antibodies as primary antibodies: rabbit polyclonal anti-H3K4me1 (C15410194, Diagenode, Toyama, Japan), anti-H3K4me2 (#39141, Active Motif, Carlsbad, CA, USA), anti-H3K4me3 (#07-473, Millipore, Burlington, MA, USA), anti-H3K9me2 (#1220, Abcam, Cambridge, UK), rabbit polyclonal anti-histone H3 (#1791, Abcam), anti-LSD1 (#2139, Cell Signaling Technology, Danvers, MA, USA), anti-MYC (#5605, Cell Signaling Technology), anti-Nestin (ABD69, MERCK, Darmstadt, Germany), anti-SOX2 (ab97959) anti-PARP (#9542, Cell Signaling Technology), and

mouse monoclonal anti-β-actin (#3700, Cell Signaling Technology). HRP-linked anti-rabbit IgG (#7074, Cell Signaling Technology) and HRP-linked anti-mouse IgG (#7076, Cell Signaling Technology) were used as secondary antibodies. Some images were acquired from the same membrane. Initially, the membrane was incubated with a specific antibody. After image acquisition, the membrane was thoroughly washed with TBST buffer, and the antibodies were stripped using Western BLoT Stripping Buffer (Takara, T7135A). Following additional washes, the membrane was incubated with a different antibody.

The density of bands was quantified using ImageJ software (https://imagej.nih.gov/ij/).

Animal experiments- magnetic resonance imaging (MRI)

When mice were scanned with MRI, mice were anesthetized with isoflurane (3% in air) and placed in MRS 3017 Benchtop MRI Systems (MR Solutions, Guildford, UK). Using MRI images, tumor volumes were obtained by compiling tumor areas from all slices that contained tumors and calculated by ImageJ software (https://imagej.nih.gov/ij/) {Towner, 2013 #1463}.

A

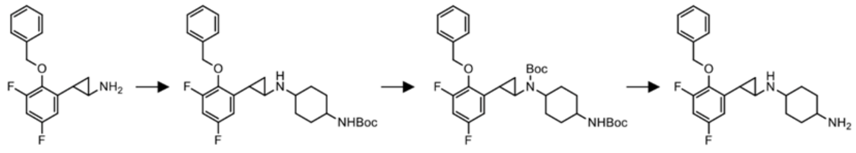

S2101 Intermediate A S2172

B

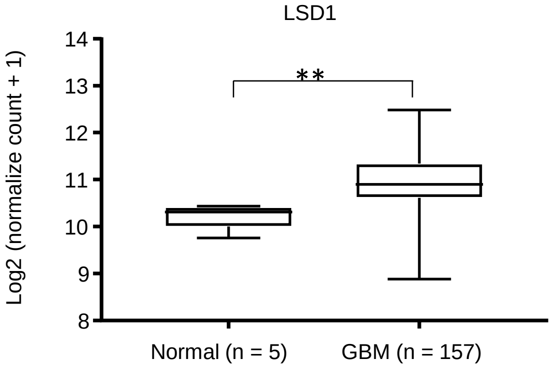

D

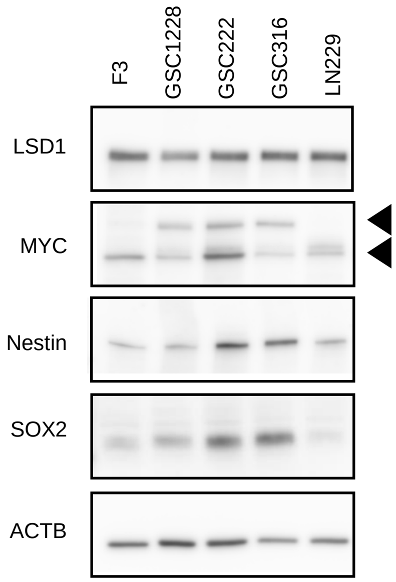

C

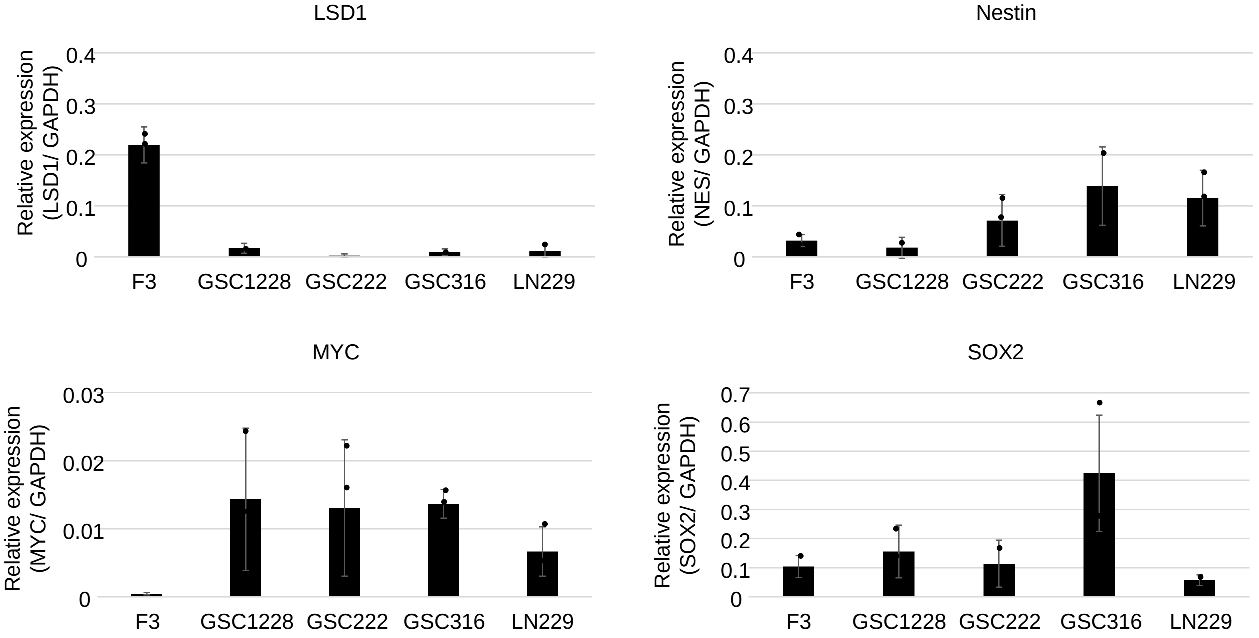

E

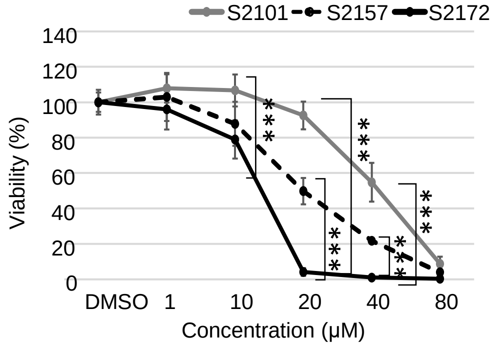

F

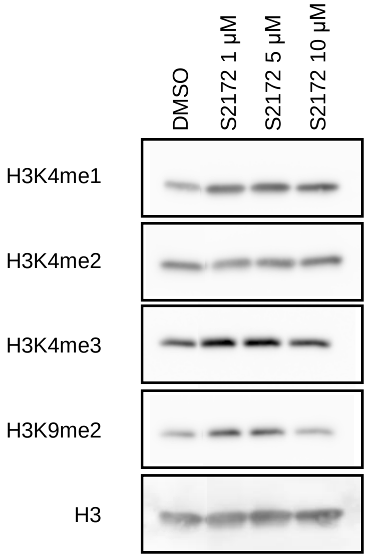

G

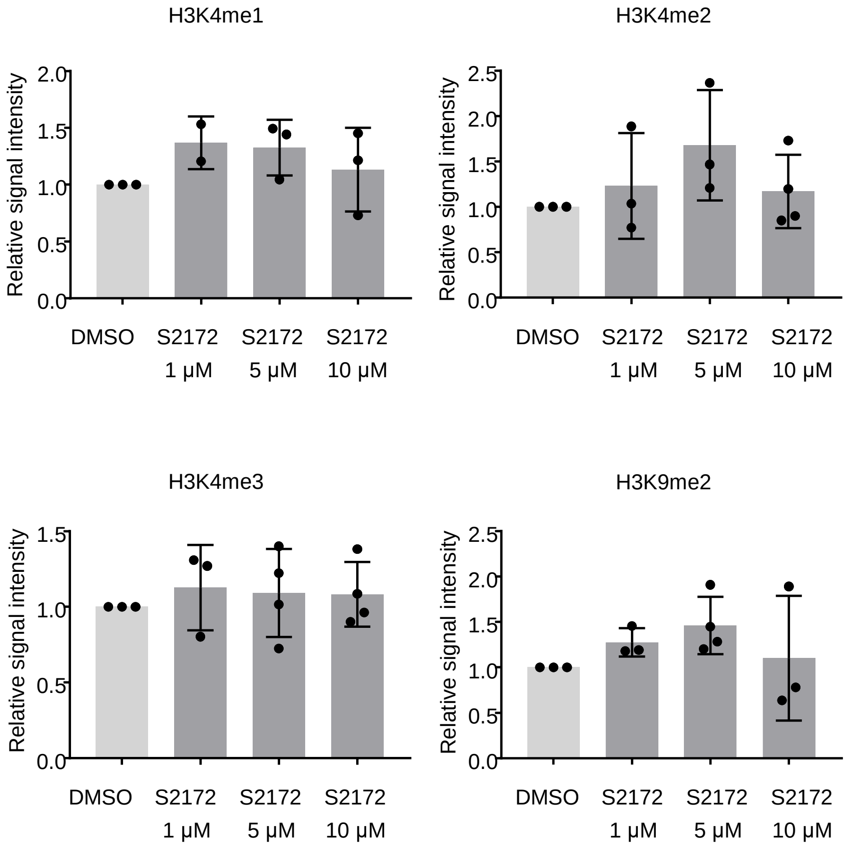

Supplementary Fig. S1

A. The scheme of the synthesis of S2172. B. TCGA data was obtained from UCSC Xena. The y-axis indicates Log2 normalized count +1 of LSD1. **, $P < 0.01$.

C.mRNA expression of LSD1 in brain tumor cell lines and normal neural stem cell F3. The y-axis indicates the relative expression to GAPDH. Error bar indicates the SD. n = 3. D. Western blot analysis of LSD1,MYC, NES, SOX2,and ACTB in same cells in C. ACTB was used as loading control. For MYC, two bands (arrow heads) were detected as MYC{Tao, 2002 #1453}. E. Inhibitory effect of LSD1 inhibitors in GSC1228. The x-axis indicates concentration of inhibitors (µM) and the y-axis indicates cell viability (%). . ***, $P < 0.001$, two-way ANOVA tests. F. GSC1228 was treated with different concentration of S2172 (1 µM, 5 µM, 10 µM) for 96 hours. Western blot analysis was performed on the treated cells to assess the levels of H3K4me1, H3K4me2, H3K4me3, and H3K9me2. G. Quantification of band signal intensities from the Western blot in Supplementary Fig. S1F. The y-axis in the panel represents the relative signal intensity of each protein, normalized to histone H3. Error bars represent the SD; n = 3.

A LN229

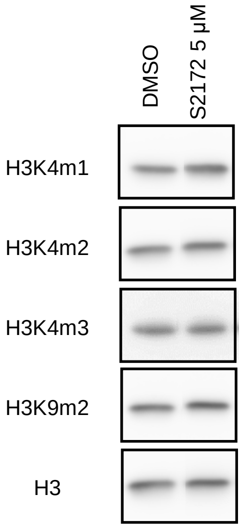

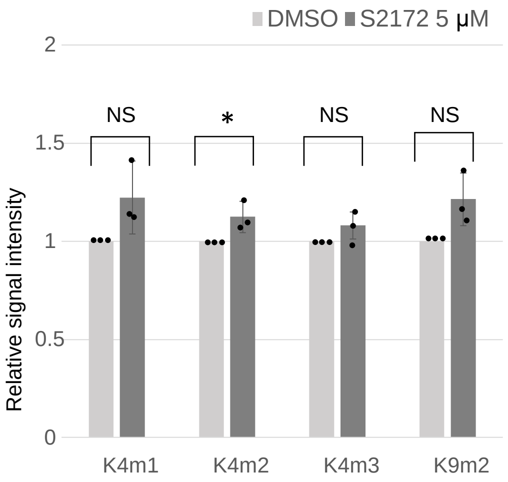

LN229

B

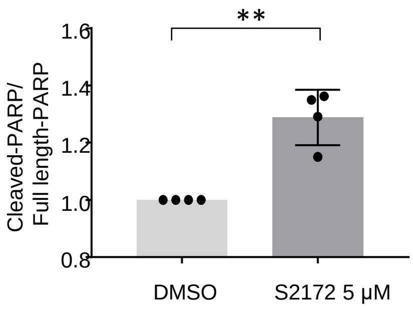

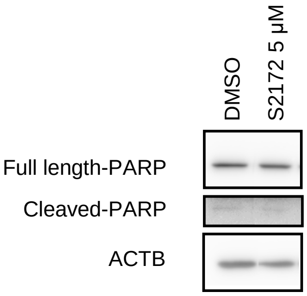

Supplementary Fig. S2
A. Western blot analysis (left panel) of H3K4me1, H3K4me2, H3K4me3, and H3K9me2 after treatment with either DMSO or S2172 5µM for 96 h in LN229. Histone H3 was used as loading control. Quantification of signal intensity of western blot (right panel). The y-axis in the panel indicates relative signal intensity of each protein normalized to histone H3. Error bars indicate the SD. n = 3. B. Left panel, PARP cleavage ratio after treatment with 5 µM of S2172 in LN229 cells. Ratio was calculated by dividing cleaved band intensity by total protein band intensity. Error bar indicates the SD. **, P<0.01. In the right panel, the full length PARP band (106 kDa) and cleaved PARP band (89 kDa) are shown. β-actin (ACTB) was used as a loading control; n = 4.

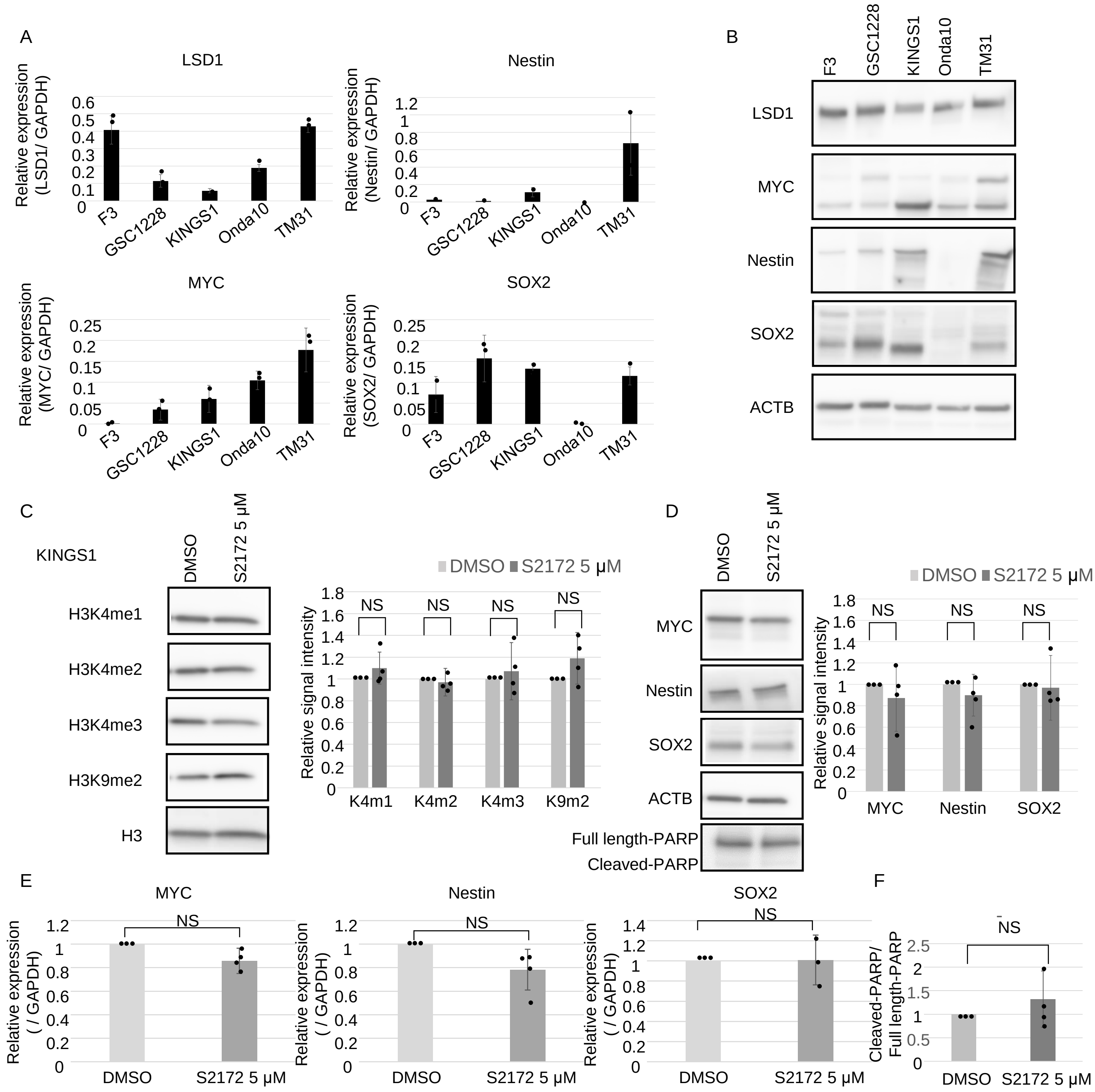

Supplementary Fig. S3

A. mRNA expression of LSD1,MYC, Nestin, SOX2,and ACTB in F3, GSC1228 and grade III glioma cells KINGS1, Onda10, and TM31. B. Western blot analysis of LSD1,MYC, Nestin, SOX2,and ACTB in same cells as shown in panel A. ACTB was used as a loading control. C. Western blot analysis (left panel) of H3K4me1, H3K4me2, H3K4me3, and H3K9me2 after treatment with either DMSO or S2172 5μM for 96 h in KINGS1 cells. Histone H3 was used as a loading control. Quantification of signal intensities of the Western blot (right panel). The y-axis represents the relative signal intensity of each protein normalized to histone H3. Error bars represent the SD; n = 4. D. Left panel: Western blot analysis of MYC, Nestin, SOX2 and PARP after treatment of KING1 with either DMSO or S2172 (5 μM) for 96 h. β-actin (ACTB) was used as a loading control. Right panel: Quantification of band signal intensities from the Western blot. Error bars represent the SD; n = 4. E. mRNA expression of stem cell marker genes following 96 h treatment of KING1 cells with S2172 (5 μM). The y-axis indicates the expression change relative to DMSO-treated cells. Error bars represent the SD; n = 3. F. PARP cleavage ratio after treatment of KING1 cells with S2172 (5 μM). The ratio was calculated by dividing the cleaved PARP band intensity by the full-length PARP band intensity. Error bars represent the SD; n = 4.

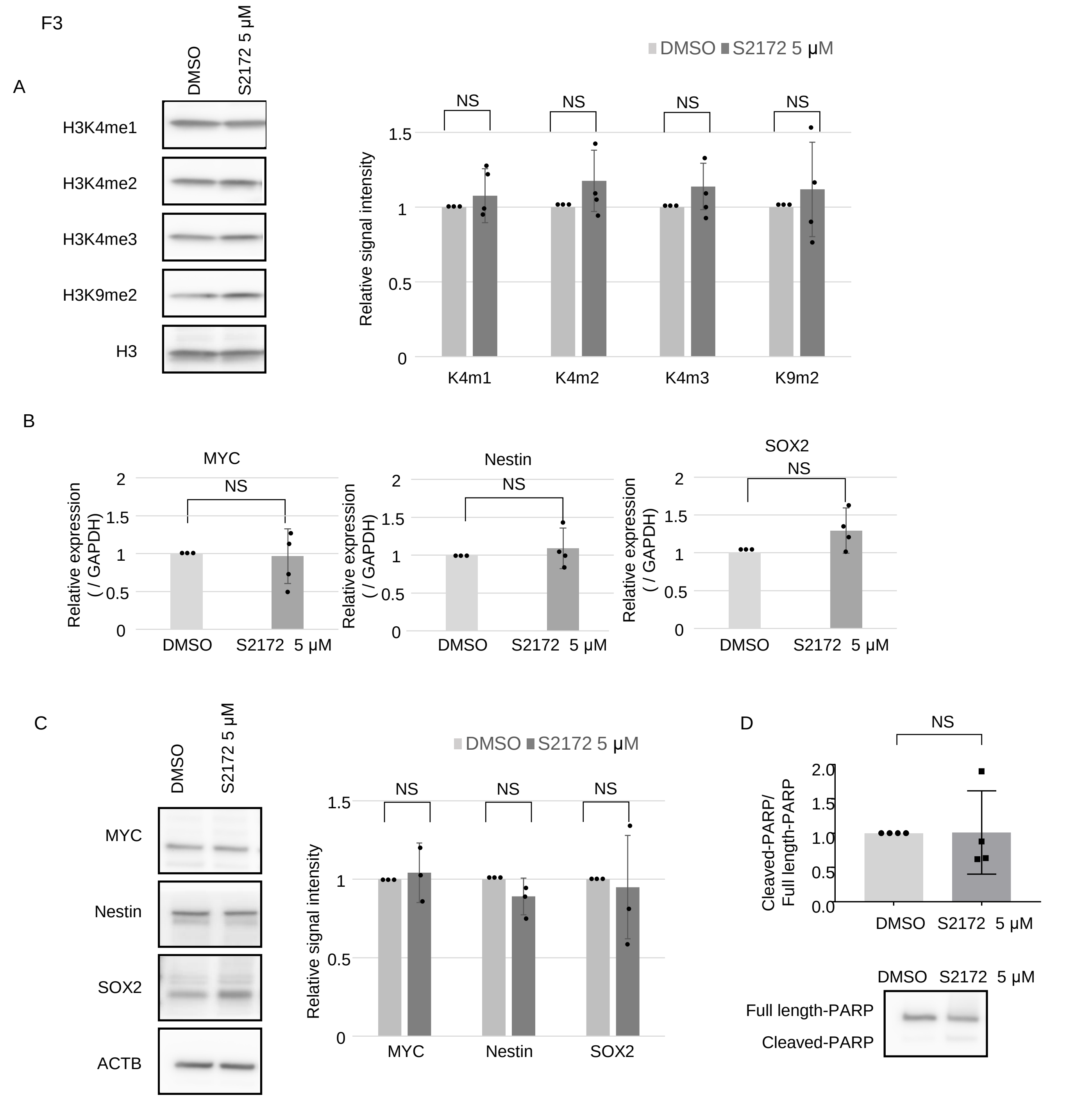

Supplementary Fig. S4

A. Left panel: Western blot analysis of H3K4me1, H3K4me2, H3K4me3, and H3K9me2 after treatment with either DMSO or S2172 5μM for 96 h in normal neural stem cell F3. Histone H3 was used as a loading control. Right panel: Quantification of signal intensities of the Western blot. The y-axis represents the relative signal intensity of each protein normalized to histone H3. Error bars represent the SD; n = 3. B. mRNA expression of stem cell marker genes following a 96 h treatment of F3 cells with 5 μM S2172. The y-axis represests the expression change relative to DMSO-treated cells. Error bars indicate the SD; n = 4. C. Left panel: Western blot analysis of MYC, Nestin, and SOX2 after treating of F3 cells with either DMSO or 5 μM S2172 for 96 h. β-actin (ACTB) was used as a loading control. Right panel: Quantification of band signal intensities from the Western blot. Error bars indicate the SD; n = 3. D. Upper panel: PARP cleavage ratio after treating F3 cells with 5 μM S2172. The ratio was calculated by dividing the intensity of the cleaved PARP band by the full-length PARP band. Error bars represent the SD; n = 4. Lower panel: Representative Western blot results of RAPR after treatment.

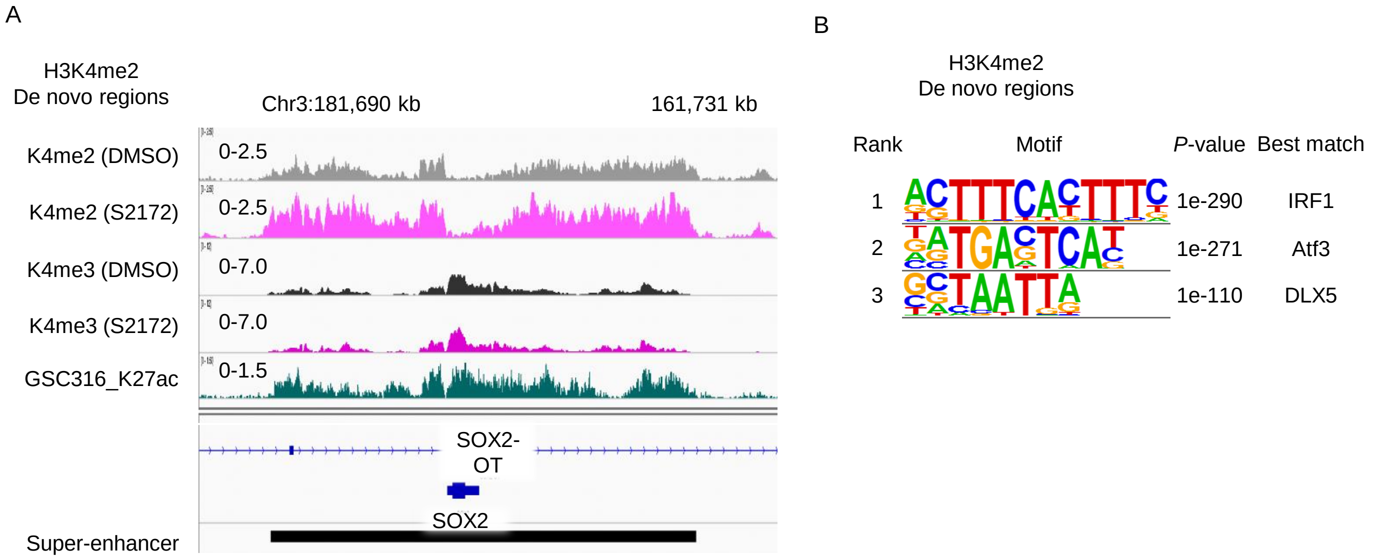

Supplementary Fig. S5

A. Representative ChIP-seq peaks at the MYC and SOX2 regions.

B. Motif enrichment at the H3K4m2 “de novo” region.

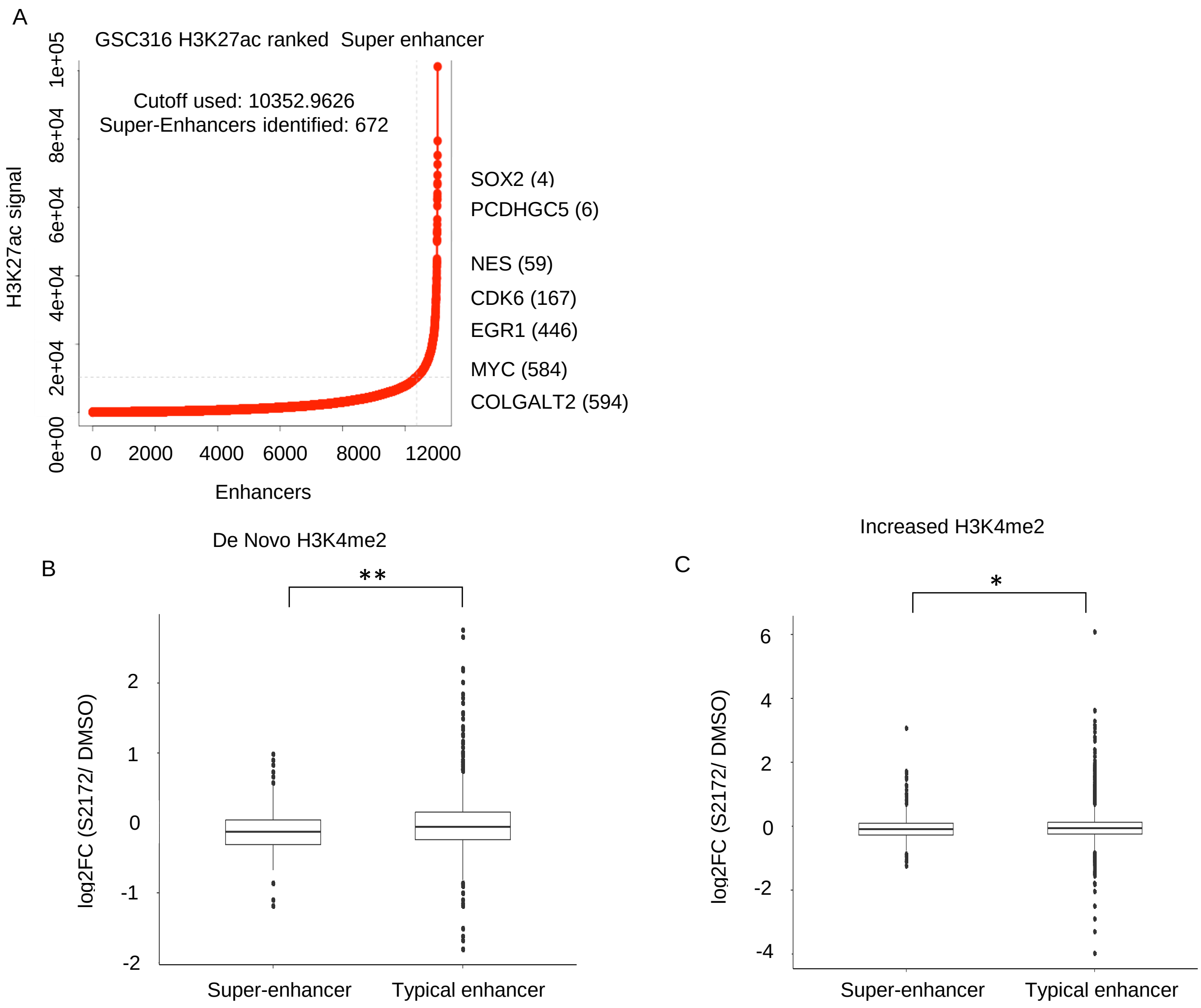

Supplementary Fig. S6
A. Super-enhancers were ranked by H3K27ac signal level by the ROSE algorithm. Signals with extremely high signals were defined as super-enhancers. B. The box plot showing the expression levels of super-enhancer associated genes and typical enhancer associated genes in de novo H3K4me2 region in GSC1228. **, $P < 0.01$, *, $P<0.05$.

A

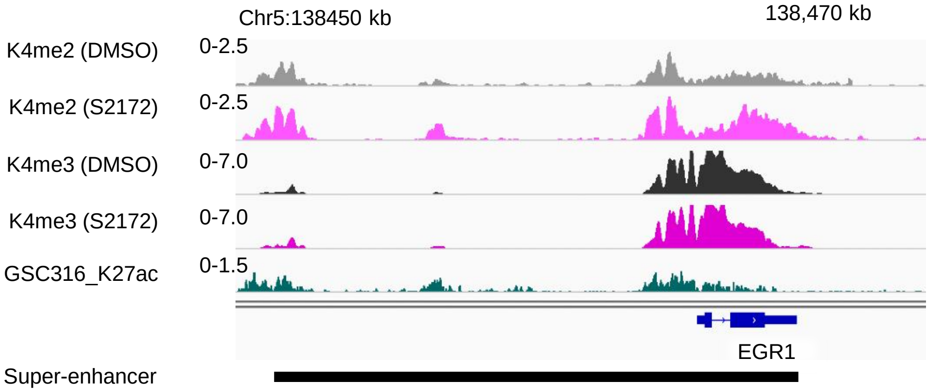

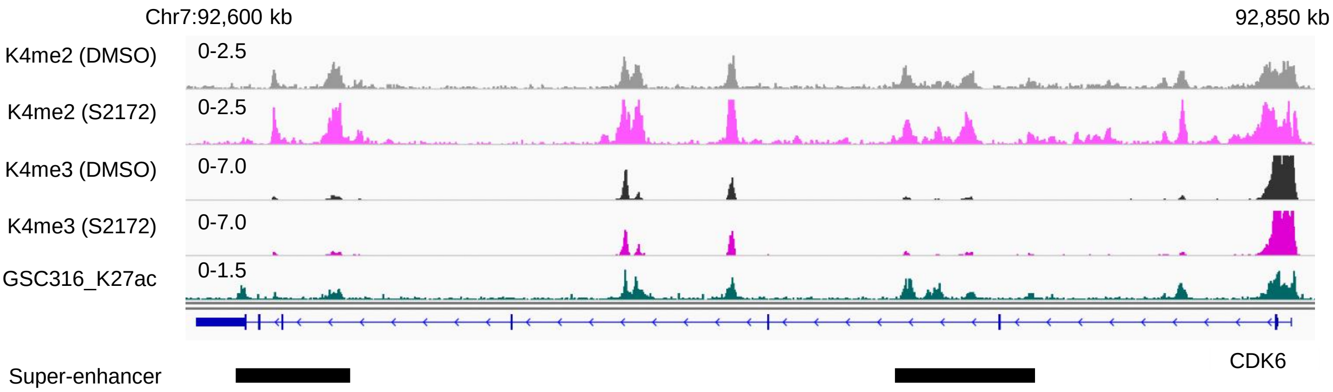

B

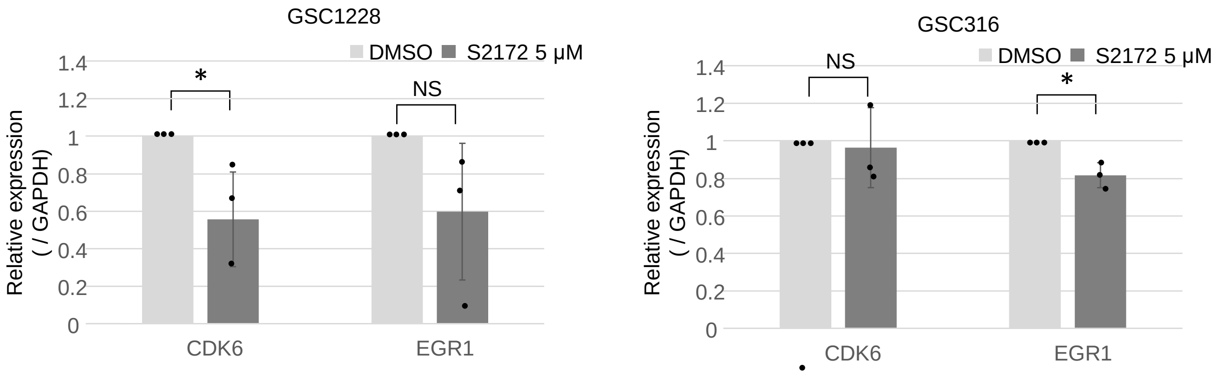

Supplementary Fig. S7

A. Representative ChIP-seq peaks at EGR1 and CDK6 (super-enhancer associated gene).

B. mRNA expression of super-enhancer regulated gene in de novo H3K4me2 peaks following 96 h treatment with S2172 in GSC1228 and GSC316. The y-axis indicates the relative expression change to DMSO treated cells. Error bar indicates the SD. *, $P < 0.05$. n = 3.

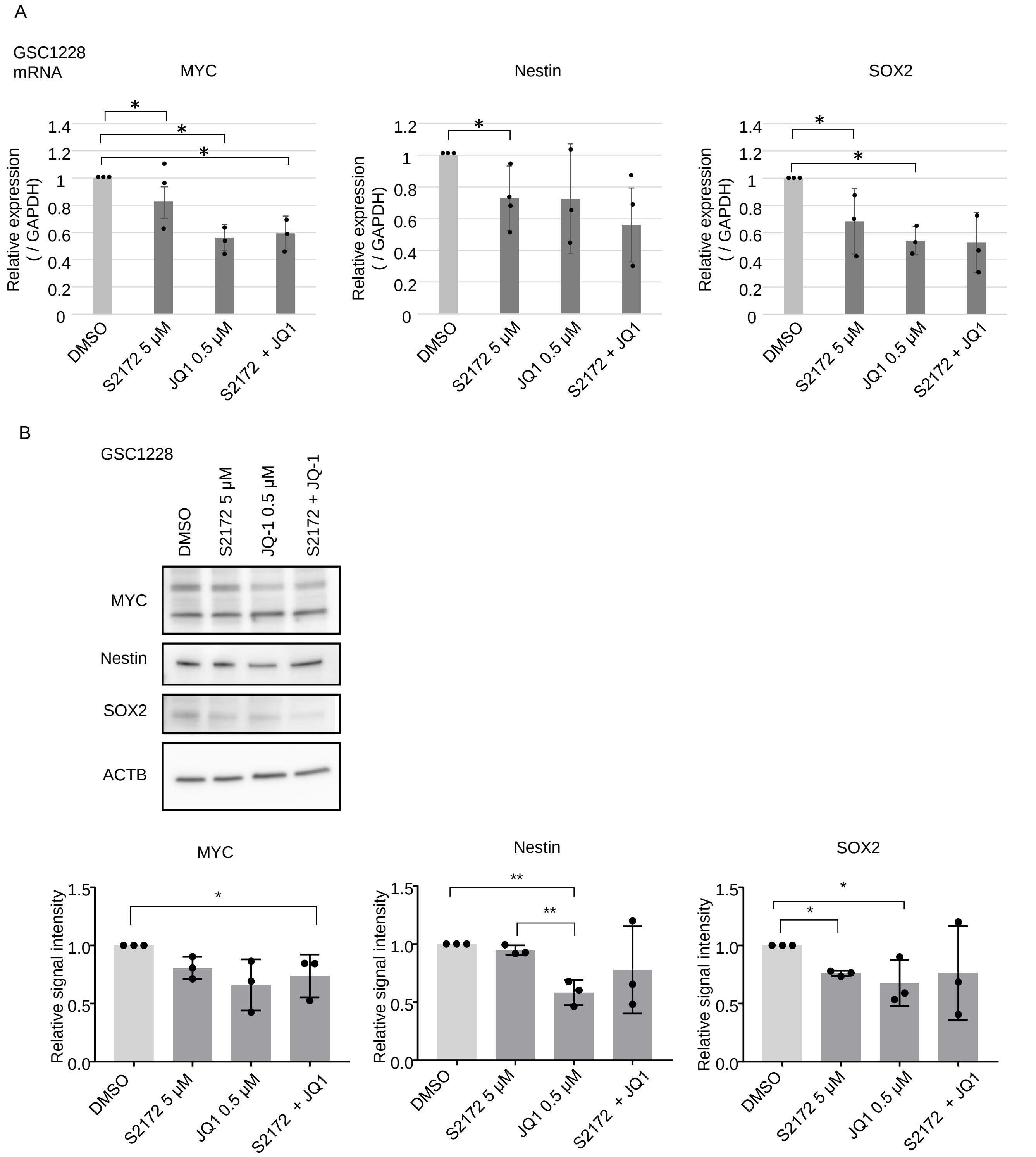

Supplementary Fig. S8

A. mRNA expression of stem cell marker genes following a 96-hour treatment of GSC1228 cells with either DMSO, 5 μM S2172, 0.5 μM JQ-1 or their combination. The y-axis represents the expression change relative to DMSO-treated cells. Error bars represent the SD; n = 3. *, *P*<0.05. B. Upper panel: Western blot analysis of MYC, Nestin, and SOX2 after a 96-hour treatment of GSC1228 cells with either DMSO, 5 μM S2172, 0.5 μM JQ-1 or their combination. β-actin (ACTB) was used as a loading control. Lower panel: Quantification of band signal intensities from the Western blot. n = 3. *, *P*<0.05, **, *P*<0.01.

A

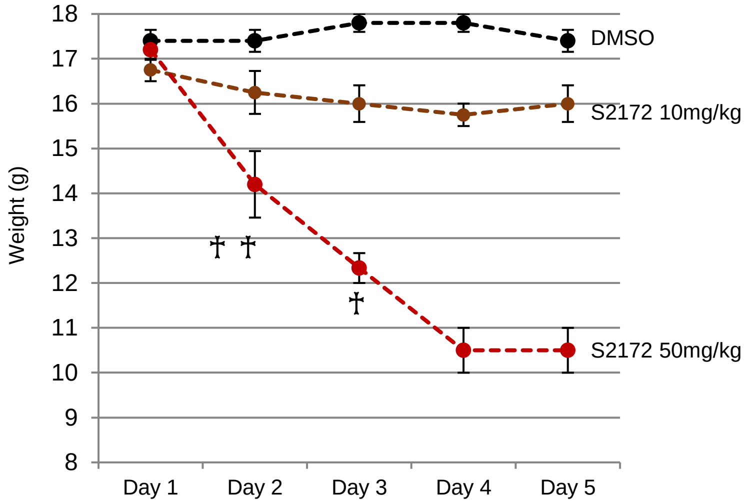

B

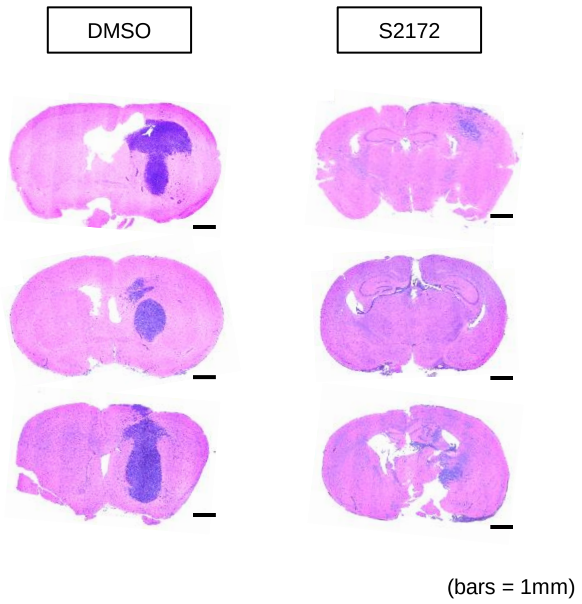

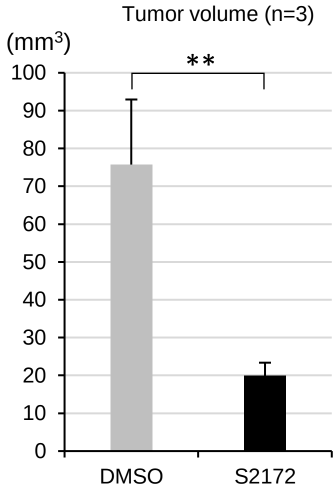

Supplementary Fig. S9

A. Determination of the maximum tolerated doses of S2172 in NOD/SCID mice.

We injected S2172 at the indicated doses into NOD/SCID mice for five consecutive days (n = 5). † indicates death of mouse. The X-axis indicates day of treatment and the Y-axis indicates the average weight of mice in each group.

B. Left, brains were resected from each mouse after treatment. Representative HE-stained whole brain section. Scale bars, 1 mm. Right, tumor volume was measured (width2 × length)/2. Error bars indicate the SD. *, P<0.05.

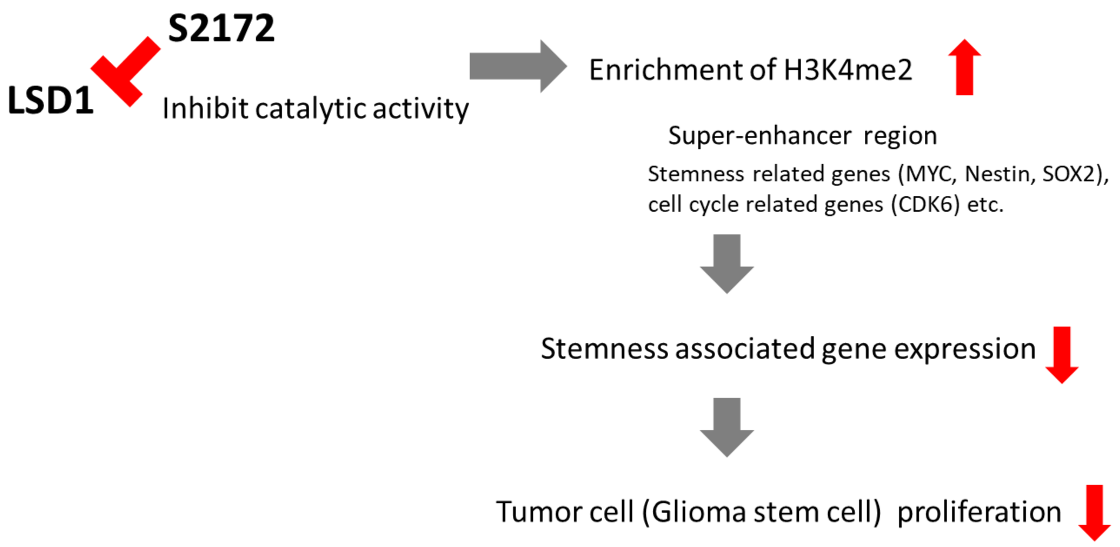

Supplementary Fig. S9
Summary of function of S2172.
LSD1 inhibitor S2172 affects histone modifications, including H3K4me1, H3K4me2, H3K4me3, and H3K9me2. Notably, the enrichment of H3K4me2 in super-enhancer region leads to the supression of gene expression. Stemness-related genes, such as MYC, Nestin, SOX2, which are regulated by super-enhancers, may be impacted by S2172. The dysregulation of these genes by S2172 is a key mechanism underlying its anti-tumor function.

Supplementary Table S1. Primer sequence

Gene name	Sequence
LSD1-F	ACTTCAGGAGTTGGAAGCGA
LSD1-R	GAGTTGAGAGAGGTGTGGCA
Nestin-F	CTGGCGGTGGCTCCAA
Nestin-R	GGTACGGGCCTGGAGGAAT
SOX2-F	AAGAAAGGGAGAGAAGTTTGAGCC
SOX2-R	GGCTCCGCGAGGAAAATC
MYC-F	GCTGCTTAGACGCTGGATTT
MYC-R	CACCGAGTCGTAGTCGAGGT
GFAP-F	ACCTCGGCACCCTGAGGCAG
GFAP-R	CCAGCGACTCAACCTTCCTC
CDK6-F	TCTGATTACCTGCTCCGCGA
CDK6-R	CTCCTCGAAGCGAAGTCCTC
COLGALT2-F	GGTGGTTTTCCCGGAGTCG
COLGALT2-R	TCAGTGGCTGCCCAGATG
EGR1-F	CACCTGACCGCAGAGTCT
EGR1-R	CAAGGTGTTGCCACTGTTGG
PCDHGC5-F	GCTCTAATACGCTGCGGGAG
PCDHGC5-R	CTCTGGGCCTGAGAGAAACG

Supplementary Table S2. *In vitro* enzyme inhibition parameters of S2172

Enzyme	k_{inact} [s^{-1}]	Ki [μM]	k_{inact}/Ki [M^{-1} s^{-1}]
LSD1	0.0026 ± 0.0002	0.27 ± 0.06	9,800 ± 570
LSD2	ND	> 250	ND
MAO-A	ND	> 250	ND
MAO-B	ND	> 250	ND

Means ± SE (N = 3). ND, not determined.
